# Crosstalk between CCL7 and CCR3 promotes metastasis of colon cancer cells via ERK-JNK signaling pathways

**DOI:** 10.18632/oncotarget.9209

**Published:** 2016-05-06

**Authors:** Yeo Song Lee, So-Young Kim, Su Jeong Song, Hye Kyung Hong, Yura Lee, Bo Young Oh, Woo Yong Lee, Yong Beom Cho

**Affiliations:** ^1^ Samsung Biomedical Research Institute, Sungkyunkwan University, Seoul, Republic of Korea; ^2^ Department of Surgery, Samsung Medical Center, Sungkyunkwan University School of Medicine, Seoul, Republic of Korea; ^3^ Department of Health Sciences and Technology, Samsung Advanced Institute for Health Sciences and Technology, Sungkyunkwan University, Seoul, Republic of Korea; ^4^ Department of Medical Device Management & Research, SAIHST, Sungkyunkwan University, Seoul, Republic of Korea

**Keywords:** CCL7, CCR3, ERK-JNK signaling, metastasis, colon cancer

## Abstract

Chemokine ligand 7 (CCL7) enhances cancer progression and metastasis via epithelial-mesenchymal transition (EMT). However, little is known about the molecular mechanism of CCL7-induced EMT signaling cascade in colon cancer. Thus, the objective of this study was to investigate CCL7-induced EMT signaling pathway and its role in the progression and metastasis of colon cancer. To demonstrate the effect of CCL7 on EMT induction, HCT116 and HT29 cells overexpressing CCL7 were generated. CCL7-induced EMT and its downstream signaling pathway were evaluated by both *in vitro* and *in vivo* experiments. In *in vitro* studies, CCL7 was found to interplay with CC chemokine receptor 3 (CCR3), resulting in enhanced cellular proliferation, invasion, and migration via ERK and JNK signaling pathway. To validate these findings, we established ectopic and orthotopic mouse models injected with CCL7-overexpressed cells. In ectopic mouse models, we observed that CCL7-overexpressed cells grew significantly faster than control cells. In orthotopic mouse models, we found that liver and lung metastasis developed only in mice injected with CCL7-overexpressed cells. This study is the first one focusing on the EMT cascade via CCL7-CCR3-ERK-JNK signaling axis in colon cancer. Our novel findings will improve our understanding on the mechanism of metastatic process and provide potential therapeutic strategies for preventing metastasis in colon cancer.

## INTRODUCTION

Colorectal cancer (CRC) occurs when tumors form in the lining of the large intestine. Cells with abnormal growth have the ability to invade or spread to other parts of the body (metastasis). Metastasis is the most common cause of death in CRC patients. Activation of epithelial-mesenchymal transition (EMT) increases metastasis [1–3]. Thus, it is important to understand the mechanisms of EMT to increase the survival rate of CRC. It has been suggested that chemokine (C-C motif) ligand (CCLs) can attract tumor cells by interacting with CC chemokine receptors (CCRs). Their interplay enhances angiogenesis, tumorigenesis, host-tumor connection [4–6], and EMT [7, 8]. CCL7 is a small cytokine known as chemokine previously called monocyte chemotactic protein-3 [9]. It is produced by certain tumor cell lines, fibroblast, colonic epithelial cells, and macrophages [10–13]. CCL7 can enhance the invasion and migration of prostate cancer [14], oral squamous cell carcinoma [7], and gastric cancer [15]. CCL7 is expressed higher in liver metastasis compared to that in their corresponding primary CRC tissues of patients [16]. These clinical studies suggest that elucidating the mechanism of CCL7 related EMT is important for our understanding on the progression of CRC.

CCL7 activates immune cells via binding to CCR1, CCR2, CCR3, and CCR5 [17, 18]. Some of these interplays are highly associated with tumor cell metastasis [19]. CCR3 is increased in inflammatory cells that have infiltrated Hodgkin's lymphoma [20]. CCR3 is also associated with higher grades of malignancy in renal cell carcinoma [21], malignant cutaneous tumor [22], and glioblastoma [23]. More recently, it has been demonstrated that a prostate cancer cell line also expresses CCR3 and that the overexpression of CCR3 is significantly associated with cancer cell metastasis [14]. These chemokine and chemokine receptor communications will activate mitogen-activated protein kinases (MAPKs) signaling pathways involved in various cellular processes, including cell proliferation, differentiation, and migration [24–28]. Mammals express at least three distinctly groups of MAPK signaling molecules, including extracellular signal-related kinases (ERK-1/2), Jun amino-terminal kinases (JNK1/2/3), and p38 proteins [29–32]. Recent studies have shown that the downstream pathways of MAPK cascades are activated by CCL7 [33]. However, little is known about the EMT process mediating through CCL7-CCR3-MAPK signaling axis. Therefore, the objective of this study was to investigate how complex network of CCL7-CCR3 would influence the tumor progression and metastasis in terms of MAPK signaling pathways using both *in vitro* and *in vivo* approaches so that we could suggest strategies for preventing colon cancer cell metastasis involving CCR3 antagonists.

## RESULTS

### Effect of CCL7 on colon cancer cell proliferation

**Figure 1:**
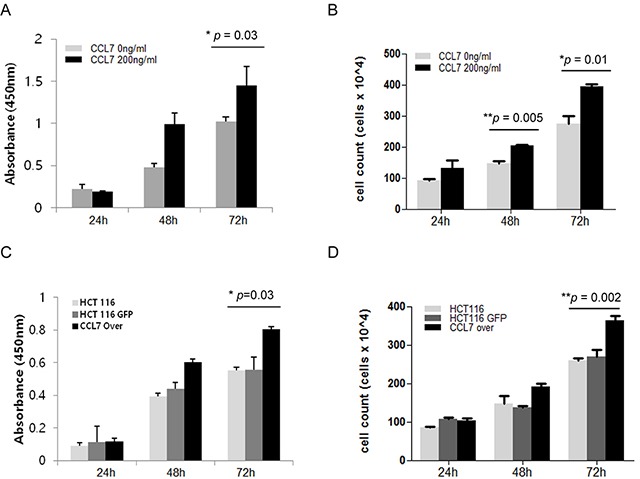
CCL7 induces cell proliferation in HCT116 cells Cell proliferation of HCT116 cells was evaluated by **A.** WST-1 indirect assay or **B.** Cell counting (direct method) using a hemocytometer and trypan blue staining at 24, 48, and 72 hours with or without recombinant CCL7 (200 ng/ml). **C-D.** The same experiment was carried out in HCT116 cells overexpressing CCL7 or GFP (control). Both experiments were performed in parallels in triplicates. Results shown are mean value ± SE. *P < 0.05; **P < 0.01.

To determine whether CCL7 has direct effect on the proliferation of colon cancer cells, we performed both WST-1 assay (indirect method) and cell counting assay (direct method) for HCT116 cells. Treatment with recombinant CCL7 for 48 and 72 hours enhanced cell proliferation compared to untreated control cells in both WST-1 assay (Figure 1A) and cell counting analysis (Figure 1B). Overexpression of CCL7 in HCT116 cells also induced cell proliferation at 72 hours post transfection compared to GFP-expressing control cells in both WST-1 assay (Figure 1C) and cell counting analysis (Figure 1D). These results highlight that CCL7 can effectively induce proliferation of colon cancer cells.

### CCL7 increases the expression of chemokine receptor CCR3 in HCT116 and HT29 cells

**Figure 2:**
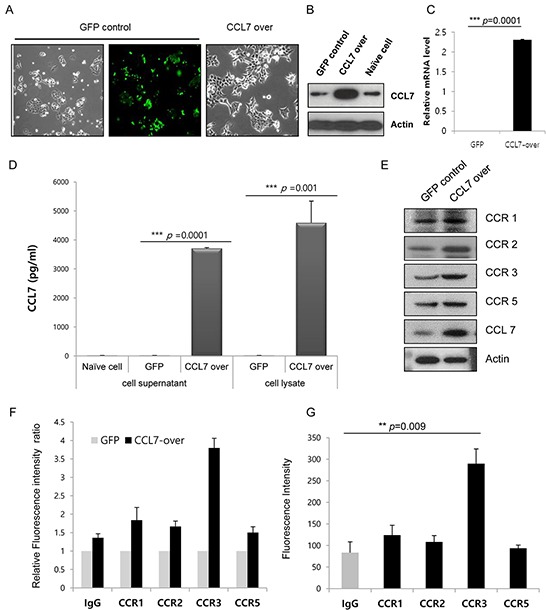
CCL7 increases expression of chemokine receptor CCR3 **A.** CCL7 overexpression induces morphological changes in HCT116 cells. Representative images of cells taken at 400× magnification are shown. **B.** Total cell lysates were subjected to western blot analysis to confirm CCL7 overexpression. Actin was used as a loading control. **C.** Transcriptional levels of *CCL7* were measured using real-time PCR. *β-actin* expression was used as an internal control to obtain the relative quantification of gene expression. **D.** CCL7 secretion was measured by multiplex magnetic immunoassay of HCT116 cell lysates and supernatants. Expression patterns of CCR1, -2, -3, and -5 protein were monitored with **E.** Western blot and **F, G.** FACS analysis in CCL7 overexpressing (E, F) or CCL7 recombinant protein treated HCT116 cells (G). Columns: means ± SEs. **P < 0.01; ***P < 0.001.

To investigate the role of CCL7 in colon cancer cells, we established HCT116 and HT29 cell line that stably overexpressed CCL7 by lentiviral transduction. The morphology of CCL7 overexpressing cells was changed compared to that of control GFP-expressing cells. Mesenchymal phenotypes such as loss of cell polarity, spindle-like cell shape, and loss of cell-to-cell adhesion were distinct in CCL7 overexpressing cells, whereas epithelial features such as close cell-to-cell adhesion were still observed in GFP expressing control cells (Figure 2A). CCL7 overexpression following lentiviral transduction was confirmed by western blot (Figure 2B; Supplementary Figure S1A) and real-time PCR analysis (Figure 2C). Measurement of CCL7 secretion by multiplex magnetic immunoassay of HCT116 cell lysates and supernatants showed that CCL7 secretion level was increased in CCL7 overexpressing cells compared to that of control GFP expressing cells (Figure 2D).

To investigate the effect of CCL7 overexpression on CCR expression, we examined the expression levels of CCR1, CCR2, CCR3, and CCR5 in stable GFP/CCL7 transfected HCT116 cells by western blot and FACS analyses. We found that the expression of CCR3 was increased higher than that of CCR1, CCR2, or CCR5 in both CCL7 overexpressing cells (Figure 2E and 2F) and cells treated with recombinant CCL7 (Figure 2G). We also found that the expression of CCR3 was influenced by CCL7 in HT29 cells (Supplementary Figure S1A and S1B). Hence, we chose CCR3 as a responsible receptor for CCL7 in this study. Taken together, our data indicate that CCL7 can significantly stimulate CCR3 expression in colon cancer cells.

### CCL7 promotes migration and invasion of HCT116 and HT29 cells via CCR3

**Figure 3:**
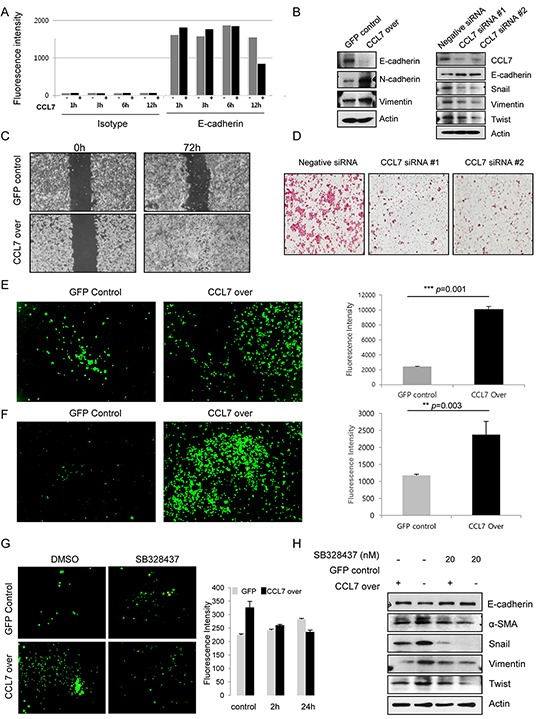
CCL7 induces migration and invasion of colon cancer cells via CCR3 **A.** Quantitation of E-cadherin expression on the surface of HCT116 cells treated with or without recombinant CCL7 (200 ng/ml) using FACS analysis. **B.** Expression of E-cadherin, N-cadherin, and vimentin in HCT116 cells stably transfected with GFP or CCL7 were measured by western blotting (left panel). Expression of CCL7 and EMT markers in negative siRNA control-treated or CCL7 specific siRNAs-treated HCT116 cell extracts (right panel). Actin was used as a loading control. **C.** A wound healing assay was performed by creating a wound on a confluent monolayer of stable GFP/CCL7 overexpressing cells using l-Dish 35-mm high culture inserts. **D.** Transwell matrigel invasion assays of HCT116 cells after transfection with 100 nM of negative siRNA control or CCL7 specific siRNAs. **E.** Cell migration and **F.** Invasion was measured using a trans-well migration chamber (left panels). Mean fluorescence intensity (MFI) of invaded area is presented in bar graphs (right panels). **G.** HCT116 cells were treated with 20 nM CCR3 inhibitor SB 328437. Representative images of invaded cells are shown (left panels). MFI values are presented in bar graphs (right panels). **H.** Expression of EMT markers in HCT116 cells stably transfected with GFP or CCL7 with or without treatment with 20 nM SB 328437 (CCR3 inhibitor) was measured by western blotting. Actin was used as a loading control. Columns: means ± SEs. ** *P* < 0.01; *** *P* < 0.001.

Loss of E-cadherin expression on the cell membrane enables cancer cell migration and invasion. To explore the function of CCL7 in colon cancer motility and invasiveness, we analyzed E-cadherin expression on the surface of HCT116 cells treated with or without recombinant CCL7 using FACS analysis. Our results revealed that treatment with recombinant CCL7 induced loss of E-cadherin (Figure 3A). Next, we examined the expression of E-cadherin, vimentin, and N-cadherin in stable CCL7 overexpressing HCT116 cells and HT29 cells by western blot analysis. As expected, E-cadherin expression was decreased in both CCL7 overexpressing HCT116 and HT29 cells, whereas N-cadherin and vimentin expression was increased compared to that in the cells transfected with control vector (Figure 3B, left panel; Supplementary Figure S1A). In addition, CCL7 dose-dependently decreased E-cadherin expression in HT29 cells (Supplementary Figure S1B). Furthermore, inhibition of CCL7 expression by siRNAs markedly decreased master EMT transcription factors such as snail and twist that repress epithelial-specific gene expression and influenced the expression of E-cadherin and vimentin (Figure 3B, right panel).

Next, we conducted wound healing assay, cell migration assay, and invasion assay. Interestingly, the wound healing ability of CCL7 overexpressing HCT116 cells was markedly increased compared to that of cells expressing control GFP (Figure 3C). As anticipated, siRNAs of CCL7 decreased the invasion of HCT116 cells (Figure 3D), which correlated with results of western blot analysis. Trans-well migration chamber analysis data using calcein staining showed that the migration and invasion abilities of CCL7 overexpressing cells were increased more than 2-fold compared to those of controls (Figure 3E and 3F). Accordingly, we treated HCT116 and HT29 cells with CCR3 inhibitor SB328437 for the indicated times to elucidate whether CCR3 could induce CCL7-EMT process. Our results revealed that CCL7-induced invasive capacity and motility were decreased after treatment with 20 nM SB328437 in HCT116 (Figure 3G) and HT29 cells (Supplementary Figure S1C).

To corroborate this CCR3 dependence, we examined protein expression of E-cadherin and alpha smooth muscle actin (α-SMA), snail, vimentin, and twist in HCT116 cells treated with or without SB328437 by western blot analysis. As predicted, the expression of EMT markers was altered in the presence of CCR3 inhibitor SB328437 (Figure 3H). These compelling results reveal that CCR3 is a major activator of CCL7 induced cell invasion and migration, the major characteristics of EMT in colon cancer cells.

### Involvement of CCR3 in CCL7-induced ERK and JNK activation

**Figure 4:**
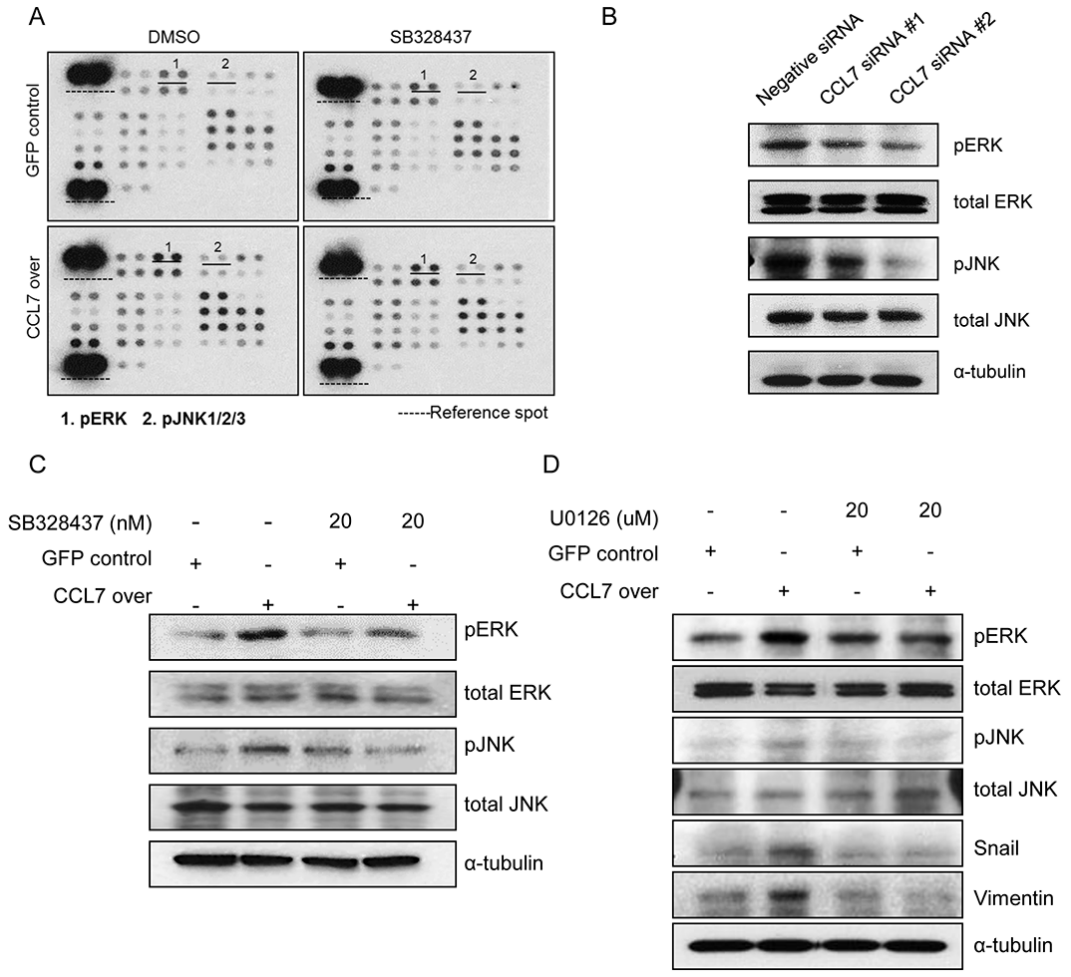
Involvement of CCR3 in CCL7-induced ERK and JNK activation **A.** Protein phosphorylation array in HCT116 cells stably transfected with GFP/CCL7 with or without treatment with 20 nM SB 328437 (CCR3 inhibitor). **B.** Expression of phosphorylated and total ERK/JNK in negative siRNA control-treated or CCL7 specific siRNA-treated HCT116 cell. **C.** Western blot analysis of phosphorylated and total ERK/JNK in HCT116 cells stably transfected with GFP/CCL7 with or without treatment with 20 nM SB 328437. **D.** Expression of phosphorylated and total ERK/JNK and EMT markers in HCT116 cells stably transfected with GFP or CCL7 with or without treatment with 20 uM U0126 (ERK/JNK inhibitor) was measured by western blotting. Actin was used as a loading control.

To further explore the role of CCR3 in EMT-related pathways inducedby CCL7, we investigated the effect of CCR3 inhibitor SB328437 on CCL7 overexpressing HCT116 cells. We conducted a proteome profiler phospho-kinase array to determine which signaling pathways were affected the most by CCL7 through CCR3. Our results revealed that MAPK pathways, especially ERK and JNK, were activated by CCL7. These activations were inhibited by SB328437 (Figure 4A). Western blot analysis using CCL7 siRNAs confirmed that CCL7 correlated with ERK and JNK activation (Figure 4B). In HT29 cells, we obtained similar results that CCL7 was associated with ERK and JNK activation (Supplementary Figure S1A and S1B).

Western blot analysis confirmed that the phosphorylation levels of ERK and JNK were increased by CCL7 overexpression. These phosphorylation level were significantly inhibited by 20 nM SB328437 (Figure 4C). To confirm these findings, we treated HCT116 cells with U0126 to inhibit both ERK and JNK activity. Western blot results revealed that CCL7-provoked expression of EMT transcription factor snail and EMT marker, vimentin were altered by U0126 treatment (Figure 4D). These data demonstrate that chemokine receptor CCR3 activates both ERK and JNK signaling pathways during CCL7-induced EMT processes.

### CCL7 provokes tumorigenicity and metastasis *in vivo*

**Figure 5:**
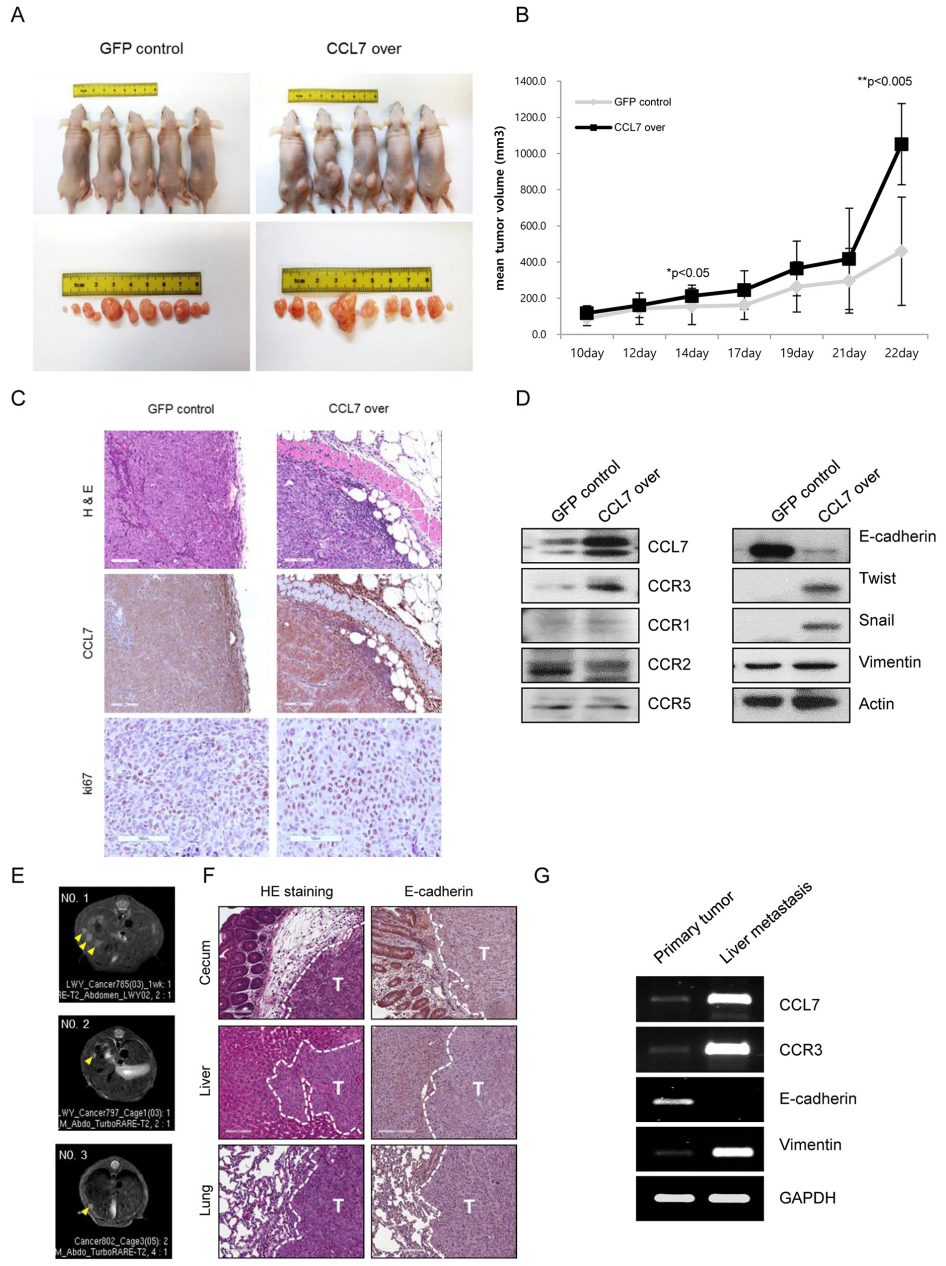
CCL7 overexpression provokes tumorigenicity and metastasis in HCT116 cells *in vivo* **A.** Tumor images and **B.** tumor volumes at 3 weeks after transplantation of HCT116 cells overexpressing GFP (control) or CCL7 into nude mice (*n* = 5). Tumor size was measured once a week with a caliper. Tumor volume was calculated using the following formula: (short length × long length × width)/2. **C.** Haematoxylin and eosin (H&E) stain of stable GFP/CCL7 overexpressing HCT116 primary tumors at 22 days after subcutaneous injection. Scale bar, 100 μm (upper panel). CCL7 (middle panel) and Ki-67 (lower panel)-stained sections of primary tumors formed by stable GFP/CCL7 overexpressing HCT116 cells. Scale bar, 100 μm. **D.** Western blot analysis for CCL7, CCR3, CCR1, CCR2, CCR5 (left panel), and EMT markers (right panel) in primary tumors formed by stable GFP/CCL7 overexpressing HCT116 cells. **E.** Magnetic resonance imaging (MRI) detection of liver metastasis (yellow arrows) in orthotopic colorectal cancer mouse models. **F.** H&E stain of primary cecal tumor (upper left), liver metastatic tumor (middle left), lung metastatic tumor (lower left), E-cadherin stain of primary cecal tumor (upper right), liver metastatic tumor (middle right), and lung metastatic tumor (lower right) isolated from mice that received orthotopic cecum injection of stable GFP/CCL7 overexpressing HCT116 cells. Scale bar, 100 μm; T, tumor; **G.** Representative RT-PCR analysis showing CCL7, CCR3 and EMT-related gene, E-cadherin, and vimentin expression in primary cecal tumor and liver metastatic tumor.

Our current data showed that CCL7 enhanced the proliferation of colon cancer cells *in vitro* (Figure 1A-1D). To confirm these *in vitro* findings, we tested whether CCL7 overexpression could enhance tumor growth *in vivo* using a mouse xenograft model. Both CCL7 overexpressing HCT116 cells and HT29 cells grew significantly faster than GFP-expressing control HCT116 and HT29 cells when they were subcutaneously transplanted into nude mice (Figure 5A and 5B, Supplementary Figure S2A, and S2B). Between weeks 1 and 3, the mean volume of CCL7 overexpressing colon cancer tumors was higher than that of the control GFP expressing colon cancer tumors (Figure 5B, Supplementary Figure S2B). The enhancement of tumor growth in nude mice by CCL7 overexpression appeared to be much more pronounced after an initial period. H&E analysis revealed that control GFP expressing HCT116 tumors were well encapsulated within fibrous capsules and non-invasive (Figure 5C, upper left panel). In contrast, tumors of CCL7 overexpressing HCT116 cells invaded into adjacent stromal tissues (Figure 5C, upper right panel). Next, we performed tissue staining with CCL7 and Ki-67 and found that the expression levels of both CCL7 and Ki67 were increased in CCL7 overexpressing HCT116 tumors (Figure 5C, middle and lower panels). To confirm these immunohistochemical results, we conducted western blot analysis for these tissues. As anticipated, loss of E-cadherin and gain of twist, snail, and vimentin were detected in subcutaneous tissues from CCL7 overexpressing HCT116 tumor (Figure 5D, right panel). Moreover, CCR3 expression was higher than that of CCR1, CCR2, or CCR5 in the CCL7 overexpressing HCT116 tumors (Figure 5D, left panel). These results are consistent with *in vitro* results obtained from the proliferation assay and analysis of the CCL7-EMT process correlated with CCR3.

A remaining issue was whether CCL7 actually would provoke metastasis *in vivo*. To answer this question, we developed an orthotopic metastatic mouse model by injecting stable GFP/CCL7 overexpressing HCT116 cells directly into the cecum wall of mice. Six nude mice were used in each group. One mouse in the CCL7 overexpressing HCT116 cells-injected group died unexpectedly within 2 days. Tumors developed at the primary cecal site in both groups. Of note, metastasis was found only in the CCL7 overexpressing HCT116 cells-injected group (Table 1). This group showed liver (60%) and lung (80%) metastasis. MRI revealed liver metastasis in three mice of the CCL7 overexpressing HCT116 cells-injected group (Figure 5E). However, no liver metastasis was detected on MRI in GFP overexpressing HCT116 cells-injected group (data not shown).

**Table 1:** Tumorigenesis rate from cecal injecctions

Group	Cell no.	Total metastasis	Liver metastasis	Lung metastasis
HCT116 GFP	1×10^6^	0/6 (0%)	0/6 (0%)	0/6 (0%)
HCT116 CCL7*	1×10^6^	4/5 (80%)	4/5 (80%)	3/5 (60%)

* One out of six animals were dead within 2 days.

We collected primary and metastatic tumor tissues and analyzed tissue histology to confirm tumor metastasis and determine the expression level of E-cadherin as a potential metastasis suppressing marker. Examination of primary and metastatic tumors by H&E staining revealed that liver and lung metastatic nodules were developed in the CCL7 overexpressing HCT116 cells-injected group (Figure 5F, left panel) but not in mice injected with GFP overexpressing HCT116 cells (data not shown). Decreased expression of E-cadherin compared to that at normal site was observed in both primary cecum and metastatic tumors (Figure 5F, right panel). RT-PCR was used to determine the mRNA levels of CCL7, CCR3, and EMT marker such as E-cadherin and vimentin. As speculated, CCL7 and CCR3 mRNA expression levels in liver metastatic tissue were significantly higher than those in primary tumor tissue. In addition, the expression of E-cadherin and vimentin mRNA showed EMT characteristics in those tissues (Figure 5G).

## DISCUSSION

In this study, we investigated how CCL7 induced tumor cell metastasis using both *in vitro* and *in vivo* approaches. First, we found that CCL7 enhanced colon cancer cell proliferation and induced both cell invasion and cell migration *in vitro*. In ectopic mouse models, we observed that CCL7-overexpressed cells grew significantly faster than control cells. In orthotopic mouse models, we found that liver and lung metastasis were developed only in mice injected with CCL7-overexpressed cells. Second, CCR3 expression was more strongly increased than CCR1, CCR2, or CCR5 following CCL7 overexpression. Moreover, induction of cell migration and cell invasion by CCL7 was correlated with CCR3. Third, we observed that CCL7 activated ERK and JNK signaling in MAPK pathways through CCR3 in colon cancer cells.

Since these metastatic cascade results are related to EMT process, we sought to investigate how the complex network of CCL7-CCR could influence the EMT phenomenon. Recent studies have revealed that CCL7 could directly induce cell proliferation *in vitro* in coronary artery smooth muscle cells and in vascular smooth muscle cells [33, 34]. In this study, we also found that CCL7 enhanced proliferation of colon cancer cells. In addition, we observed that CCR3 was the most responsible receptor of CCL7. This result is consistent with previous studies on human prostate cancer cells and oral squamous cell carcinoma in which CCL7 is closely correlated with CCR3. Previous studies have also shown that CCL7/CCR3 axis is related to increased cell motility and cell proliferation [7, 35]. Similar to these results, we also found that CCL7/CCR3 crosstalk induced EMT process in colon cancer cells. Furthermore, we discovered that these EMT cascade was mediated through ERK and JNK pathways. Previously published data have shown that ERK can enhance JNK activity through c-Jun stabilization [36]. These activation provoked cyclinD expression related to tumor cell proliferation and metastasis [36]. Based on these findings, we speculated that CCL7/CCR3 crosstalk could induce ERK and JNK activation sequentially and result in enhanced EMT process.

To confirm these *in vitro* findings, we investigated whether CCL7 could enhance tumor growth and metastasis *in vivo* using ectopic and orthotopic mouse model system. Our results revealed that CCL7 overexpression indeed correlated with cancer cell tumorigenesis and metastasis. Based on these observations *in vivo*, we conclude that CCL7 has a critical role in tumor proliferation and metastasis and this role is related to CCR3.

This study is the first one focusing on metastasis via CCL7/CCR3 crosstalk and related signaling pathways in colon cancer. We also demonstrated that inhibition of these interactions could decrease the EMT process of colon cancer cells. These novel finding strongly suggest that suppressing oncogenic communication between CCL7 and CCR3 may be a potential therapeutic strategy for preventing human colon cancer metastasis. The limitation of this study was that our experiments used only cell lines. Patient-derived tumor cells or patient-derived xenograft model will be needed in the future for preclinical experiments to closely resemble tumor clinical responses in patients.

In conclusion, our results imply that CCR3- correlated ERK-JNK activation may be a molecular link in the induction of metastasis by CCL7 in colon cancer cells. Evidence of such mechanistic interplay between CCL7/CCR3 and ERK–JNK cascade provides important insights into cell invasion and migration in colon cancer. This study may be useful for developing potential therapeutic strategies to improve the survival outcome of CRC patients.

## MATERIALS AND METHODS

### Cell cultures and viral transduction

HCT116 colorectal cancer cells were cultured with RPMI 1640 (Gibco, Grand Island, NY, USA) supplemented with 10% FBS (Gibco) and 1% penicillin-streptomycin (Gibco) in a 37°C incubator with 5% CO_2_. For lentiviral transduction, HCT116 cells (5×10^4^ cells/well in a 12-well plate) were transduced with lentivirus carrying green fluorescent protein (GFP) or CCL7 at a multiplicity of infection (MOI) of 100 in the presence of 8 μg/ml Polybrene. After incubation for 24 hours, transduced cells were selected with 1,000 μg/ml hygromycin (Sigma, St. Louis, MO, USA). GFP lentivirus was used as a negative control. Selected stable cells were maintained in a 5% CO_2_ incubator at 37°C in growth media containing 500 μg/ml hygromycin.

### Cell proliferation assay

Cell proliferation was evaluated by using two methods: WST-1 [2-(4-iodophenyl)-3-(4-nitrophenyl)-5-(2,4-disulfophenyl)-2H-tetrazolium)] (Roche, Indianapolis, IN, USA), an indirect method using colorimetric assay to determine cellular viability by measuring the metabolic conversion of a water-soluble tetrazolium salt, and cell counting, a direct method using a hemocytometer and trypan blue staining (Sigma, St Louis, MO, USA). Viability was assessed 24, 48, and 72 hours after CCL7 treatment and in CCL7 overexpressing cells. Three different experiments were performed under each experimental condition.

### Cell lysis and western blot analysis

To prepare whole cell extract, cells were lysed using Pro-prep buffer (Intron Biotechnology, Seoul, Korea) including protease inhibitors. A total of 20–60 μg of protein extract was resolved by SDS-PAGE and transferred to polyvinylidene fluoride (PVDF) membranes. The membranes were probed with primary antibodies against CCL7 (#7899F8, Genway, Sandiego, CA, US [7, 14]A), CCR1 (#3414-100, Biovision, Milpitas, CA, USA), CCR2 (#NB110-55674, Novus, Littleton, USA), CCR3 (#PRS1109, Sigma, St. Louis, MO, USA), CCR5 (#ab32048, Abcam, Cambridge, MA), E-cadherin (#610181, BD Bioscience, San Jose, CA, USA), N-cadherin (#4061, Cell Signaling Technology, Danvers, USA), vimentin (#sc-32322, Santa Cruz Biotechnology, CA, USA), Twist1(#ab50887, Abcam, Cambridge, MA), α-SMA (#SC-53142, Santa Cruz Biotechnology, CA, USA), Snail (#3895, Cell Signaling Technology, Danvers, USA), phospho JNK1/2/3 (#TA312591, Origene, Rockville, MD, USA), total JNK1/2/3 (#TA325661, Origene, Rockville, MD, USA), phospho ERK (#612358, BD Bioscience, San Jose, CA, USA), total ERK (#9102, Cell Signaling Technology, Danvers, USA), and β-actin (#3700, Cell Signaling Technology, Danvers, USA) followed by incubation with secondary antibodies conjugated to horseradish peroxidase (Santa Cruz Biotechnology, CA, USA). β-actin was used as a loading control in western blot analysis.

### Wound healing assay

Wound healing assays were performed using l-Dish 35-mm high culture inserts (Ibidi, Martinsried, Germany) according to the manufacturer's protocols. Briefly, on the day before experiment, cells were seeded into each well of culture inserts and incubated at 37°C in a humidified atmosphere supplied with 5% CO_2_. After cell attachment, culture inserts were gently removed using sterile tweezers. Cells were then incubated for 6 hours in serum-free media. Images were captured at regular intervals as cells migrated to close the wound. These images were used to quantify cell migration rate.

### Cell migration and invasion assay

Cell migration and invasion assays were carried out using uncoated trans-well migration chambers (BD Bioscience, San Jose, CA, USA) in 24-well cell culture plates. Cells (5×10^4^ /well) were loaded into the migration and invasion chambers in serum-free RPMI media. The lower chambers contained RPMI supplemented with 10% FBS as a chemoattractant. Plates were incubated for 24 or 48 hours. Cells were then stained with calcein (2 μM, BD Biosciences, San Jose, CA, USA) or hematoxylin-eosin and mounted. Cell migration and invasion was quantified by fluorescence measurements with a VICTOR2 Multilabel Counter (Perkin Elmer, Boston, MA, USA) equipped with a 485/520 nm filter set.

### Flow cytometry analysis

After treatment with 200 ng/ml CCL7 recombinant protein (#282-P3, R&D, Minneapolis, MN, USA) for 1, 3, 6, and 12 hours, cells were suspended in PBS and incubated with FcR blocking reagent (Miltenyi Biotec, Gladbach, Germany) for 10 min. Cells were stained for 30-40 min at 4°C with the following directly conjugated antibodies: anti-human E-Cadherin-APC (#180224, R&D, Minneapolis, MN, USA), anti-human CCR1 (#PA-141062, Thermo), anti-human CCR2 (#ab32144, Abcam, Cambridge, MA), anti-human CCR3 (#ab32512, Abcam, Cambridge, MA), anti-human CCR5 (#ab32048, Abcam, Cambridge, MA), and anti-human IgG-APC isotype (Miltenyi Biotec, Miltenyi, Germany). Secondary antibody for CCR1, -2, -3, and -5 was conjugated to PE (#12-4739-81, Ebioscience). IgG isotype antibodies were used in parallel as control. Cells were analyzed on an Accuri C6 flow cytometer (BD Biosciences, San Jose, CA, USA) with CFlow software (BD Biosciences, San Jose, CA, USA).

### Multiplex magnetic immunoassay

For analysis of CCL7 secretion, cell culture supernatant was used directly for analysis. Cell pellet was resuspended in lysis buffer. Lysates and supernatants of HCT116-GFP and HCT116-CCL7 overexpressing cells were analyzed for CCL7 protein expression using microsphere-based ProcartaPlex™ immunoassays (Bio-Rad, Hercules, CA, USA) with a Luminex 200 analyzer (xMAP^®^ Technology, USA) according to the manufacturers' instructions. This kit includes all non-target specific reagents, assay diluent, wash buffers, streptavidin-PE, and detection plates.

### RNA isolation and quantitative real-time PCR

Total RNA was extracted from cells transfected with CCL7 or GFP (RNAprep Mini kit, Qiagen, Venlo, Netherlands) and 500 ng of RNA was subjected to reverse transcription using MuLV reverse transcriptase (NEB, UK). Real-time quantitative PCR amplification was performed with two-step TaqMan Probe Master Mix (Roche, Indianapolis, IN, USA) or SYBR green PCR Mater Mix (Applied Biosystems, Carlsbad, CA, USA) on an ABI real-time thermocycler (Applied Biosystems, Carlsbad, CA, USA). Human-specific TaqMan PCR primer set were purchased from Applied Biosystems. *CCL7* Hs00171147_m1 and β-actin Hs01060665_g1 were used in this study. The following genes were evaluated: CCL7 gene (upstream primer: 5′-accaccagtagccactgtcc -3′; downstream primer: 5′- gaggagcatcccacagtttt - 3′); CCR3 gene (upstream primer: 5′- gacctgctcttcctcgtcac-3′, downstream primer: 5′- agcagagggagaacgagaca-3′); E-cadherin gene (upstream primer: 5′-ggtcgacaaaggacagccta-3′, downstream primer: 5′- ggcgtagaccaagaaatgga-3′); vimentin gene (upstream primer: 5′- ctcctccccctgtcacatac-3′, downstream primer: 5′-tgattggcatcaggaccgtt-3′); Gapdh gene (upstream primer: 5′-aatcccatcaccatcttcca- 3′, downstream primer: 5′-tggactccacgacgtactca-3′). The mRNA level of each gene was quantified using the ΔΔCt method and normalized to that of β-actin.

### Proteome profiler array

Total protein was prepared with protein lysis buffer in cells overexpressing CCL7 or GFP (control) and 50 μg of cell lysate was incubated overnight at 4°C according to the protocol of Proteome Profiler Human phospho-Kinase array kit (ARY003B, R&D, Minneapolis, MN, USA). The positive signal seen on developed film can be quickly identified by placing the transparency overlay on the array image and aligning it with the pairs of reference spots in the corners of each membrane. Pixel densities on developed X-ray film were measured and analyzed using a transmission mode scanner and image analysis software (Image J; Wayne Rasband, National Institutes of Health, USA).

### Animals

Six- to 8- week-old female BALB/c nu/nu mice weighing 15-18 g at the time of surgery were used. These mice were obtained from Orient Bio Group (Seoul, Korea) and maintained under specific pathogen-free conditions. This study was reviewed and approved by the Institutional Animal Care and Use Committee (IACUC) of Samsung Biomedical Research Institute (SBRI). SBRI is an Association for Assessment and Accreditation of Laboratory Animal Care International (AAALAC International)-accredited facility. It abides by the Institute of Laboratory Animal Resources (ILAR) guidelines.

### Xenograft experiments

Tumorigenesis was measured using an *in vivo* assay to evaluate the effect of CCL7 overexpression on cancer growth. Briefly, cells overexpressing CCL7 were suspended in 50 μl PBS supplemented with 50% matrigel and injected subcutaneously into the flanks of 6-week-old female BALB/c nu/nu mice (Charles River Laboratories, Wilmington, DE, USA). Tumor size was measured once a week with a caliper. Tumor volume was calculated using the following formula: (short length × long length × width)/2. Mice were sacrificed 3–4 weeks after the inoculation or as soon as a reduction in vitality was observed.

### Orthotopic mouse models

Nude mice were anesthetized with Zoletil (30 mg/kg) by intra-peritoneal injection (0.01 mL/mg). HCT116 cells transfected with CCL7 or GFP were prepared in 5×10^6^ cells/50μL HBSS (Gibco) in one injection. An incision was made on the middle of the lower abdomen for cecal injection. The cecum was picked-out and prepared cells were injected with a 30-gauge needle. To prevent tumor cell leakage and bleeding, a cotton swab was held over the site of injection for 1 minute. After the injection, the cecum was returned to the abdominal cavity and the wound was closed with 6-0 black silk.

### Imaging

Two weeks after the injection, the mouse model was imaged once per week to monitor liver metastasis with an *In Vivo* Imaging System (IVIS Spectrum, Caliper, Hopkinton, MA, USA). MRI imaging (Biospec 7T, Bruker, Fullerton, CA, USA) was initiated 14 days after cell injection and repeated every 1 week for as long as the liver metastasis was detected. T2-weighted axial MRI sections were obtained in the following settings: fast spin echo sequence with time to repetition of 1143.8 msec, time to echo of 25.8 msec, 160×170 matrix, 24.0 mm × 26.0 mm field of view, signal averaging of 12, section thickness of 1.0 mm; gap of 0 mm. Bioluminescence imaging was obtained using the *In Vivo* Imaging System. Mice were imaged for 1 min with sequential 5 sec exposures. Fluorescence was quantified using Living Image software 3.2 (Caliper Life Sciences, Hopkinton, MA, USA).

### Immunohistochemical staining

Four μm thick sections were cut from paraffin-embedded blocks using a microtome, deparaffinized with xylene, hydrated in serial dilutions of alcohol, and immersed in 3% H_2_O_2_. Following antigen retrieval in citrate buffer (pH 6.0), the tissue sections were incubated with protein blocking agent (Immunotech, Marseille, France) to block non-specific antibody binding for 10 minutes at room temperature and then incubated overnight at 4°C with primary antibody against E-cadherin (#3195, Cell Signaling Technology, Danvers, USA), CCL7 (#7899F8, Genway, Sandiego, CA, USA), or Ki67 (#sc-15402, Santa Cruz Biotechnology, CA, USA) in a humidified chamber. After washing with PBS three times, the sections were incubated with a biotinylated secondary antibody and streptavidin conjugated to horseradish peroxidase (Immunotech) for 60 minutes at room temperature followed by a PBS wash. The chromogen was developed for various minutes with liquid of 3, 3′-diaminobenzidine (Immunotech) followed by counterstaining with Meyer's hematoxylin. Slides were examined under a light microscope.

### Statistical analysis

Multiplex magnetic immunoassay, flow cytometry analysis, and some data from proliferation were analyzed using GraphPad Prism 5.0 software (GraphPad Software, Inc., La Jolla, CA, USA) applying one-way analysis of variance (ANOVA) with post hoc analysis using Bonferroni post hoc test. Other statistical analyses were performed using Sigma plot. Distributions were described in means ± SD and presented as column bar graphs. Data within each experiment were subjected to unpaired 2-tailed t tests for paired comparisons. All experiments were performed at least three times. The difference between groups was considered as statistically significant when *p* < 0.05.

## SUPPLEMENTARY MATERIALS FIGURES


