# Supplementary material for: Crosstalk between CCL7 and CCR3 promotes metastasis of colon cancer cells via ERK-JNK signaling pathways
Source: Oncotarget. 2016 May 6;7(24):36842–53. doi: 10.18632/oncotarget.9209 (PMC5095043; doi:10.18632/oncotarget.9209)
Supplement: Supplementary file 1 [file oncotarget-07-36842-s001.pdf]

SUPPLEMENTARY MATERIALS AND METHODS

Cell culture and reagents

HT29 colorectal cancer cells were cultured with RPMI 1640 (Gibco, Grand Island, NY, USA) supplemented with 10% FBS (Gibco) and 1% penicillin-streptomycin (Gibco) in a 5% CO_2 incubator at 37°C. Human recombinant CCL7 protein and anti-CCL7 antibody was obtained from R&D systems (R&D, Minneapolis, MN, USA).

SUPPLEMENTARY FIGURES

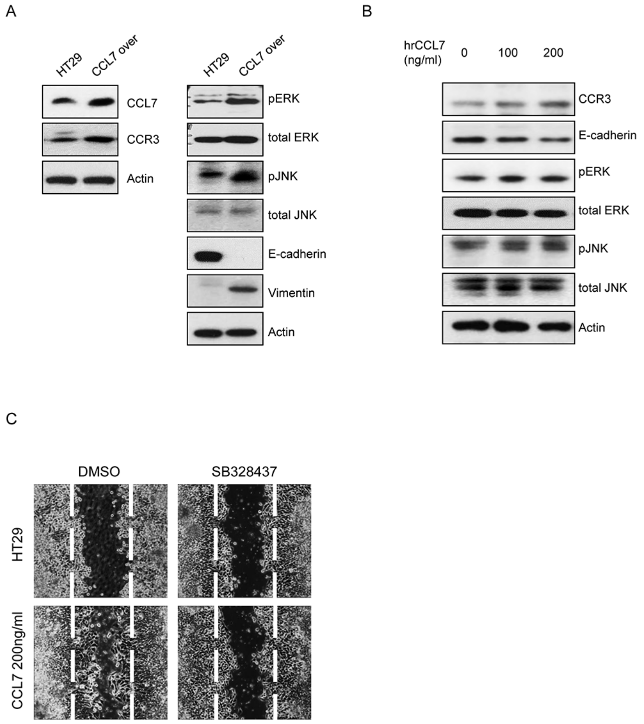

Supplementary Figure S1: CCL7 increases EMT through CCR3 in HT29 colon cancer cells. A. Expression of CCL7, CCR3 (left panel), ERK, JNK, E-cadherin, and vimentin (right panel) in HT29 cells stably transfected with GFP or CCL7 were measured by western blotting. **B.** Western blot analysis of CCR3, E-cadherin, ERK, and JNK expression in response to human recombinant CCL7 (hrCCL7) treatment for 24h. **C.** A wound healing assay was performed by creating a wound on a confluent monolayer HT29 cells. Cells were pretreated with 20 nM CCR3 inhibitor SB 328437 for 1 hour followed by incubation with hrCCL7 for 48 hours.

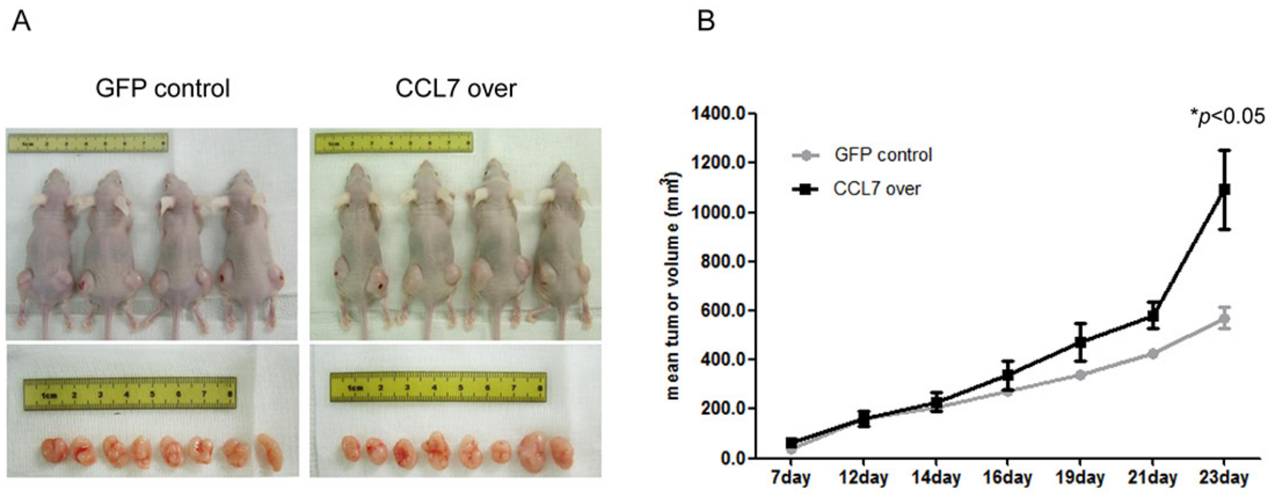

Supplementary Figure S2: CCL7 overexpression provokes tumorigenesis in HT29 cells. A. Tumor images and **B.** Tumor volumes at 3 weeks after transplantation of HT29 cells overexpressing GFP (control) or CCL7 into nude mice ($n = 4$). Tumor size was measured at least twice a week with a caliper. Tumor volume was calculated using the following formula: (short length × long length × width)/2.
